# Supplementary material for: Phosphorylation of ULK1 affects autophagosome fusion and links chaperone-mediated autophagy to macroautophagy
Source: Nat Commun. 2018 Aug 28;9:3492. doi: 10.1038/s41467-018-05449-1 (PMC6113293; doi:10.1038/s41467-018-05449-1)

Supplementary Figure 1

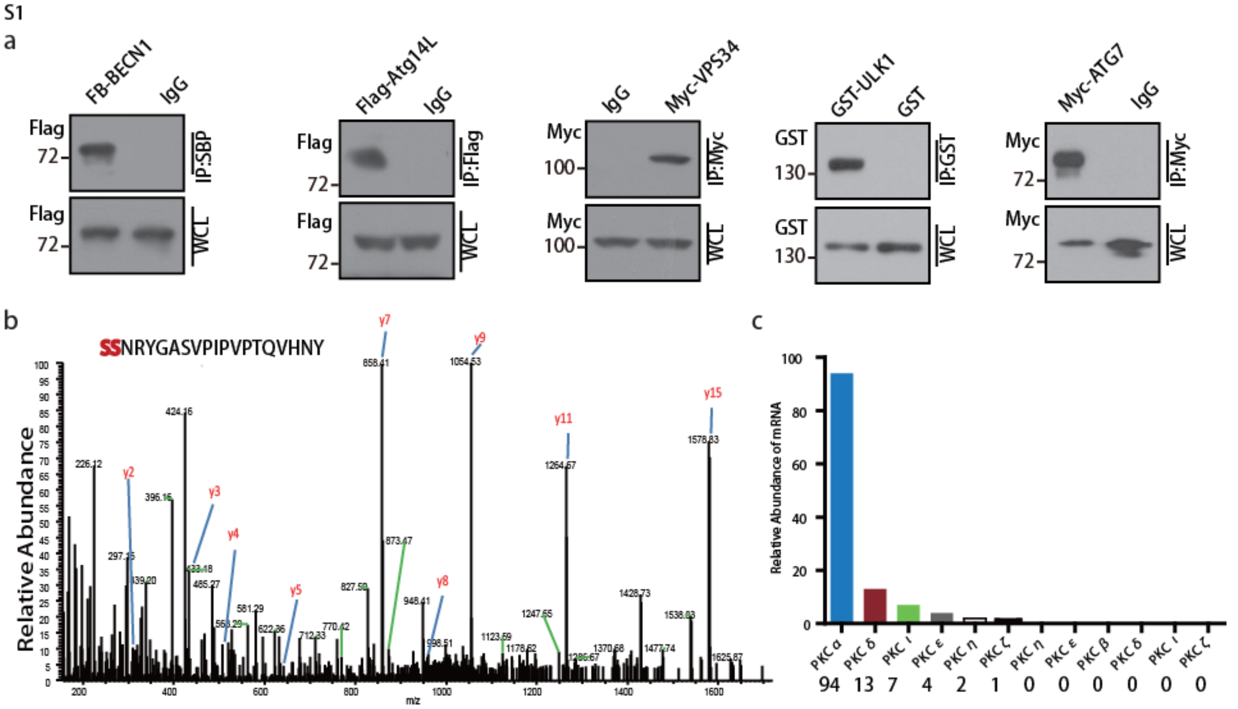

Supplementary Figure 1 PKC phosphorylates ULK1 in vitro and in vivo. (**a**) The expressions of key autophagy related proteins and the specificities of IPs. WCL: whole cell lysate (**b**) After the in vitro PKCα kinase assay, the Prokaryotic GST-ULK1 279-525 was pulled down by glutathione beads, washed for 3 times by PBS, and followed by PRM of LC-MS/MS to identify the accurate phosphorylation site. (**c**) The ratio of different PKC isoforms expression in U87 cells. The data is form Human Protein Atlas.

Supplementary Figure 2

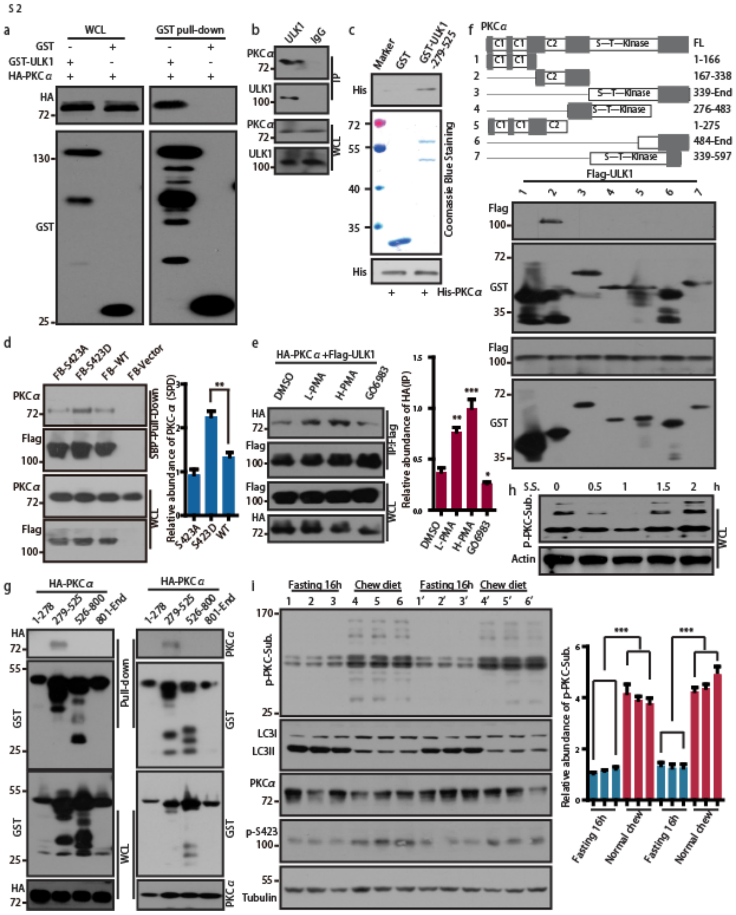

Supplementary Figure 2. PKCα physically interacts with ULK1. (**a**) HA-PKCα was cotransfected with GST-ULK1 or GST only. The pulldown was analyzed with anti-HA antibody. (**b**) Endogenous co-IP experiment for PKCα and ULK1 interaction using anti-ULK1 antibody. (**c**) Purified GST or GST-ULK1 279-525 were incubated with His-PKC α which is purified from Sf9 insect cells. After washing, the pulldown was analyzed with anti-His antibody. (**d**) FB-ULK1 variants or FB vector transfected into HEK293T cells, and the lysates were IPed with SBP beads and then detected with PKCα antibody. The IPed PKCα were quantified (**e**) Ha-PKCα was cotransfected with Flag-ULk1 and treated with DMSO, PMA (3 μM, 10 μM) and GÖ6983 for 0.5 h. The IP was performed with anti-Flag antibody and analyzed with anti-HA antibody. The IPed PKCα was quantifiedPMA:Phorbol-12-Myristate-13-Acetate; WCL: whole cell lysate (**f**) GST-tagged PKCα fragments were transfected with Flag-ULK1, Glutathione beads pulldown was performed and analyzed with anti-Flag antibody. The schematic of PKC-α truncations is in top panel. (**g**) HA-PKCα was cotransfected with GST-ULK1 fragments as in **Figure 4e**. Then the pulldown assay was performed and analyzed with indicated antibodies. (**h**) The PKC activity was determined with anti-p-PKC substrate antibody during serum starvation by WB. (**i**) The liver lysates of mice with normal chew or fasting16 hrs were analyzed with indicated antibodies. Each goup are included 3 mice. . All values are means ± SEM of three independent experiments. Student's t test (unpaired); *p<0.05, **p< 0.01, ***p<0.001. S.S.: serum starvation; WCL: whole cell lysate

Supplementary Figure 3

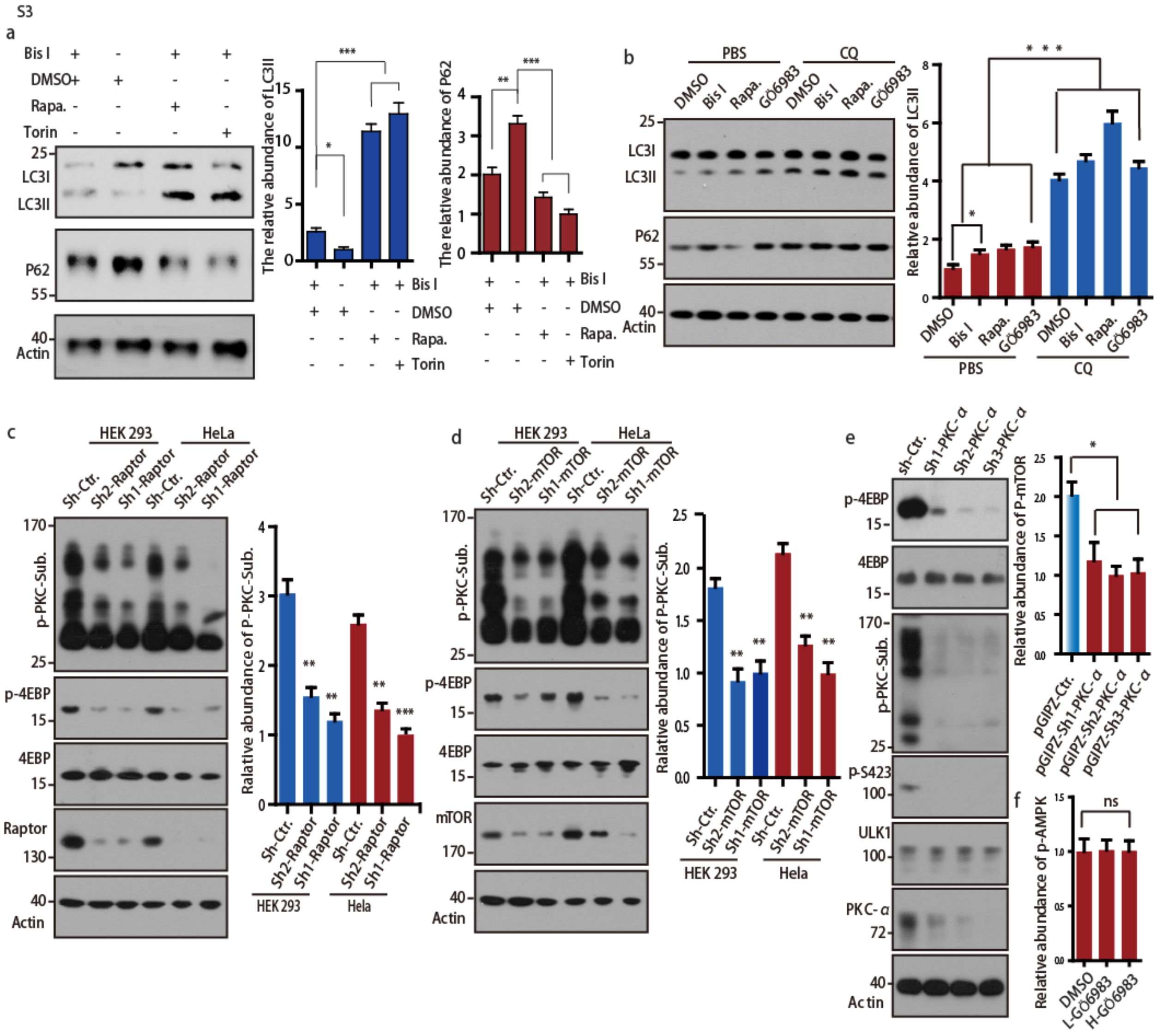

Supplementary Figure 3. PKC regulating autophagy is related to mTORC1. (**a**) HeLa cells were treated with PKC inhibitors combined with mTOR inhibitors for 0.5 h, autophagy was analyzed by WB with anti-LC3 and anti-p62 antibodies. The densities of LC-II and p62 were quantified and showed on the right panel. Bis I: Bisindolylmaleimide I; Rapa.: Rapamycin (**b**) The similar results were obtained in U87 cells as in **supplementary figure 3a**.The quantification is showed on the right panel. (**c,d**) The PKCα and mTORC1 activities were determined by anti-p-PKC substrate and anti-p-4EBP antibodies after Raptor or mTOR was knocked down in both HeLa and HEK293 cells. The PKC activities were quantified and showed on the right panels (**e**) The PKCα and mTORC1 activities were determined by anti-p-PKC substrate and anti-p-mTOR antibodies after PKCα was knocked down in HeLa. The mTOR activities were quantified and showed on the right panel. (**f**) The quantification of p-AMPK from **Figure 2f**. . All values are means ± SEM of three independent experiments. Student's t test (unpaired); ns: No significance, *p<0.05, **p< 0.01, ***p<0.001L- GÖ6983:Low concentration; H-Ö6983: High concentration

Supplementary Figure 4

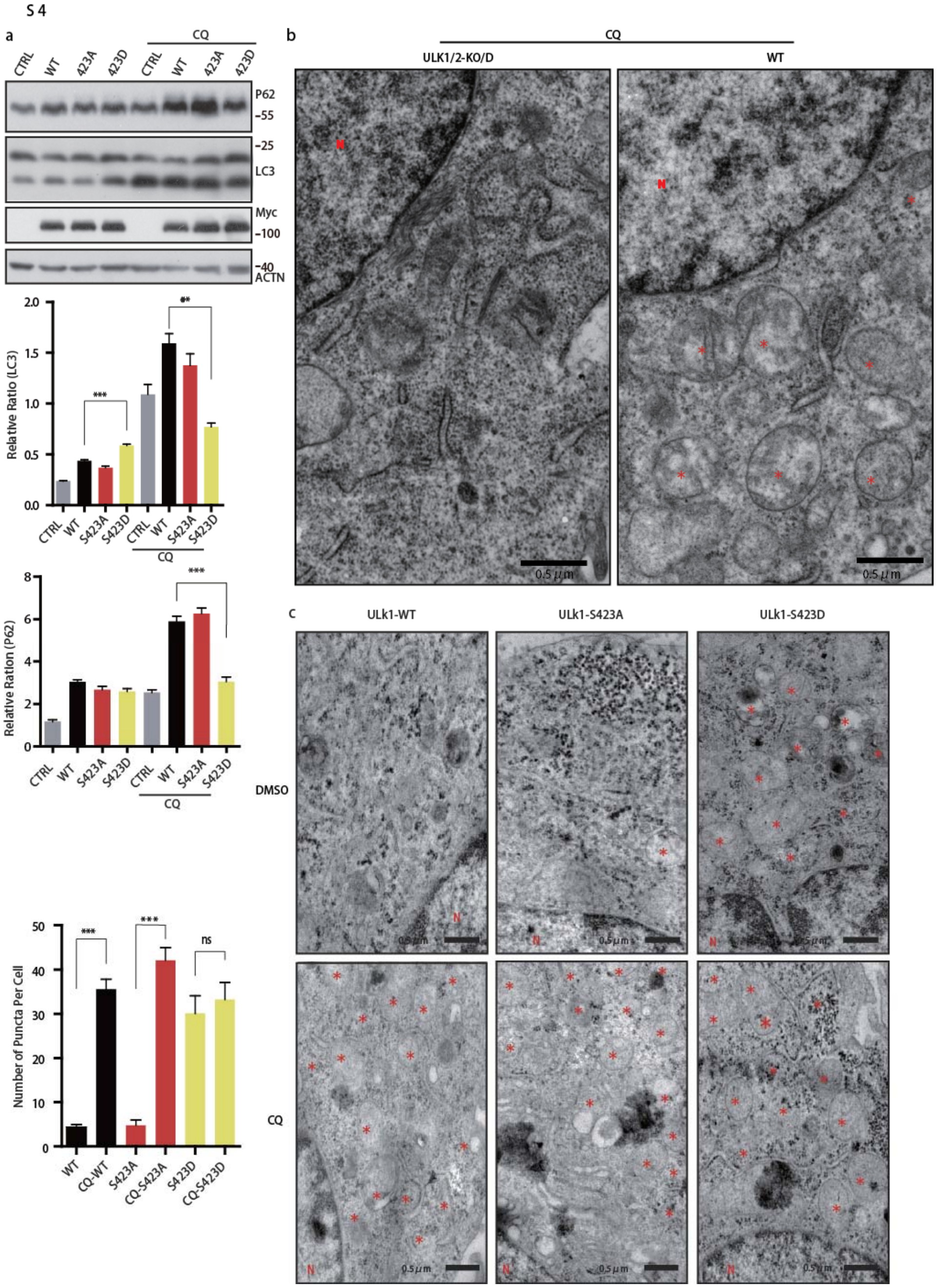

Supplementary Figure 4. Phosphorylation of ULK1 regulates autophagosome fusion. (**a**) WB analysis of LC3 and p62 in ULK1 WT and mutants overexpressing cells with/out CQ (100nM) treatment are represented as quantification of LC3-Π and p62 intensity (relative to actin). (**b**) Accumulation of complete double membrane autophagosome (red star) by TEM in ULK1/2 WT and KO/D cells with CQ treatment (100nM) (n=15). (**c**) Accumulation of complete double membrane autophagosome (red star) by TEM in ULK1 mutants stable cells in ULK1/2-KO/D cells with/out CQ(100nM) treatment (n=15). N: Nuclei. All values are means ± SEM of three independent experiments. Student's t test (unpaired); ns: No significance, *p<0.05, **p< 0.01, ***p<0.001.

Supplementary Figure 5

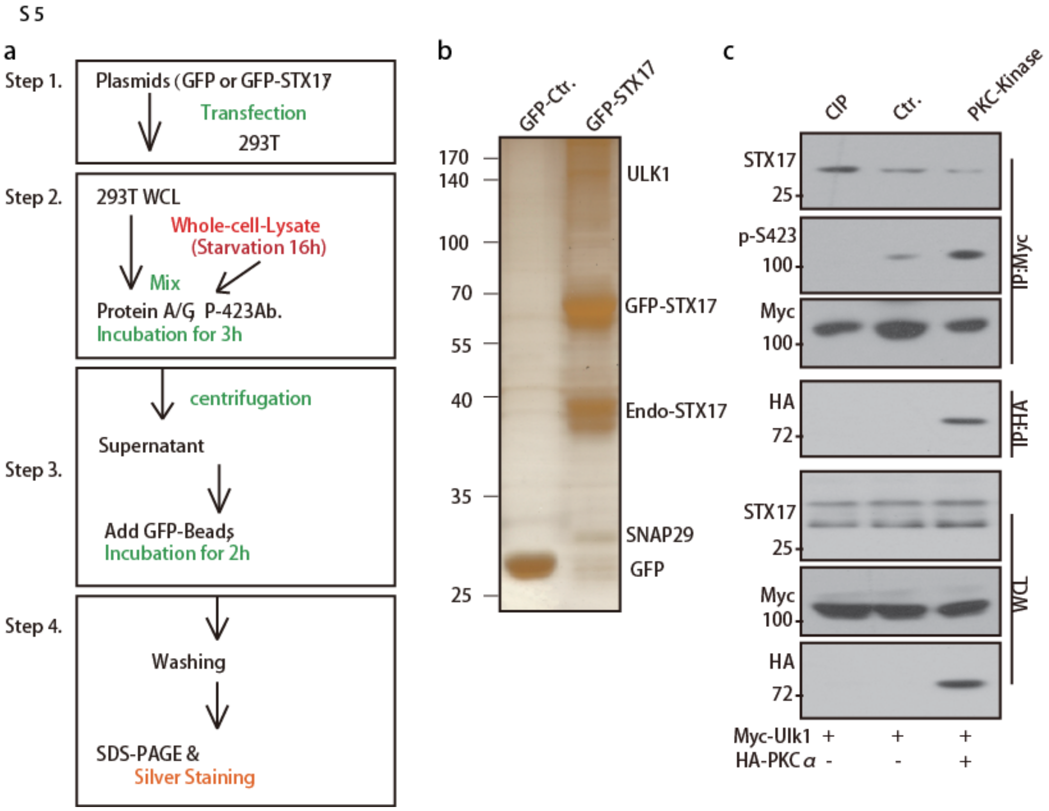

Supplementary Figure 5. STX17 and ULK1 physically interacts each other. (**a**) The steps of preparation of samples for LC-Mass using GFP-STX17. (**b**) Silver staining of STX17 coIP complex. The upper band was cut for LC-MS/MS. (**c**) The IPed Myc-ULK1 was treated with CIP or PKCα assay for 30 mins. The beads were washed and incubated with normal cell lysates for 2h, then washed again and analyzed with anti-STX17 and anti-p-S423 antibodies. WCL: whole cell lysate

Supplementary Figure 6

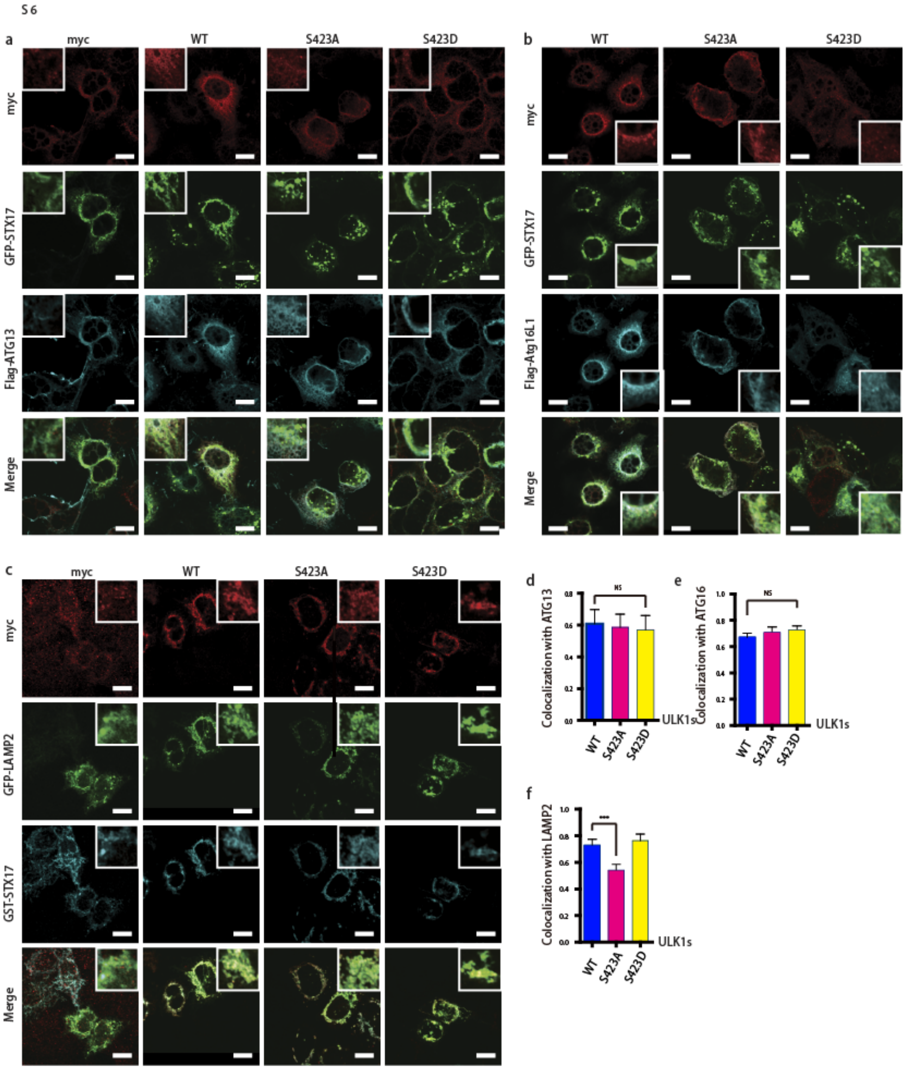

Supplementary Figure 6. Images of ULK1-STX17 colocalization with ATG13, ATG16L1 and LAMP2. (a)Representative images of HeLa cells after GFP-STX17 and Flag-ATG13 were cotransfected with Myc-ULK1 WT, S423A and S423D into Hela cells. The cells were performed immunostaining for Myc (Red), Flag (Blue) antibodies and GFP (Green) and taken the images under confocal microscope. Scale Bar, 5 µM. (**b**)Representative images of HeLa cells after GFP-STX17 and Flag-ATG16L1 were cotransfected with Myc-ULK1 WT, S423A and S423D into Hela cells. The cells were performed immunostaining for Myc (Red), Flag (Blue) antibodies and GFP (Green) and taken the images under confocal microscope. Scale Bar, 5 µM. (**c**)Representative images of HeLa cells immunostaining after GST-STX17 and GFP-LAMP2 were cotransfected with Myc-ULK1 WT, S423A and S423D into Hela cells. The cells were performed immunostaining for Myc (Red), GST (Blue) antibodies and GFP (Green) and taken the images under confocal microscope. Scale Bar, 5 µM. (**d,e,f**) The colocalization of ATG13, ATG16L1, and LAMP2a with ULK1s were measured by Pearson correlation analysis and independent-sample t-tests. The (d) represented as quantification from (a). The (e) represented as quantification from (b). The (f) represented as quantification from (c). A minimum of 20 cells. All values are means ± SEM of three independent experiments. Student's t test (unpaired); *p< 0.05, **p< 0.001.

Supplementary Figure 7

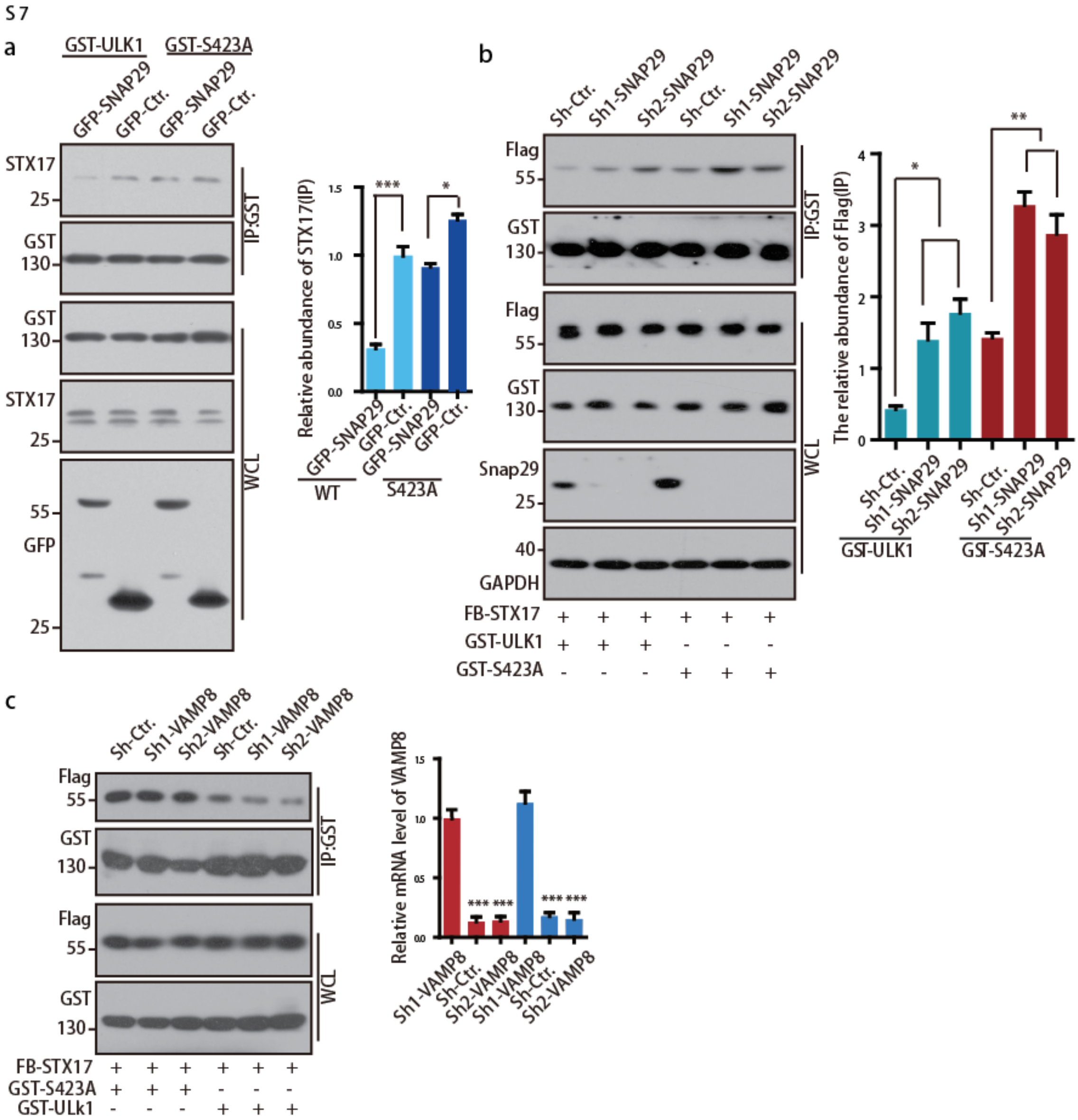

Supplementary Figure 7. Phosphorylation of ULK1 regulates STX17 complex. (**a**) Overexpression of SNAP29 decreases STX17 binding ULK1. GST-ULK1 WT, S423A were cotransfected with GFP-SNAP29 or GFP into HEK293T cells. The pulldown was detected by anti-STX17 antibody. The IPed STX17 was quantified and showed on right panel. (**b**) Knockdown of SNAP29 increases STX17 and ULK1 affinity. GST-ULK1 WT or S423A was cotransfected with FB-STX17 into HEK293 cells, then infected with shRNAs-SNAP29 lentivirus or control lentivirus. The pulldown was detected by anti-Flag antibody. The IPed STX17 was quantified on the right panle (**c**)Knockdown of VAMP8 via pGIPZ-lentivirus does not affect the interaction of ULK1 and STX17. The bond proteins were quantified on the right panel. The relative level of VAMP8 was measured by qPCR. All values are means ± SEM of three independent experiments. Student's t test (unpaired); *p<0.05, **p<0.01, ***p<0.001. WCL: whole cell lysate

Supplementary Figure 8

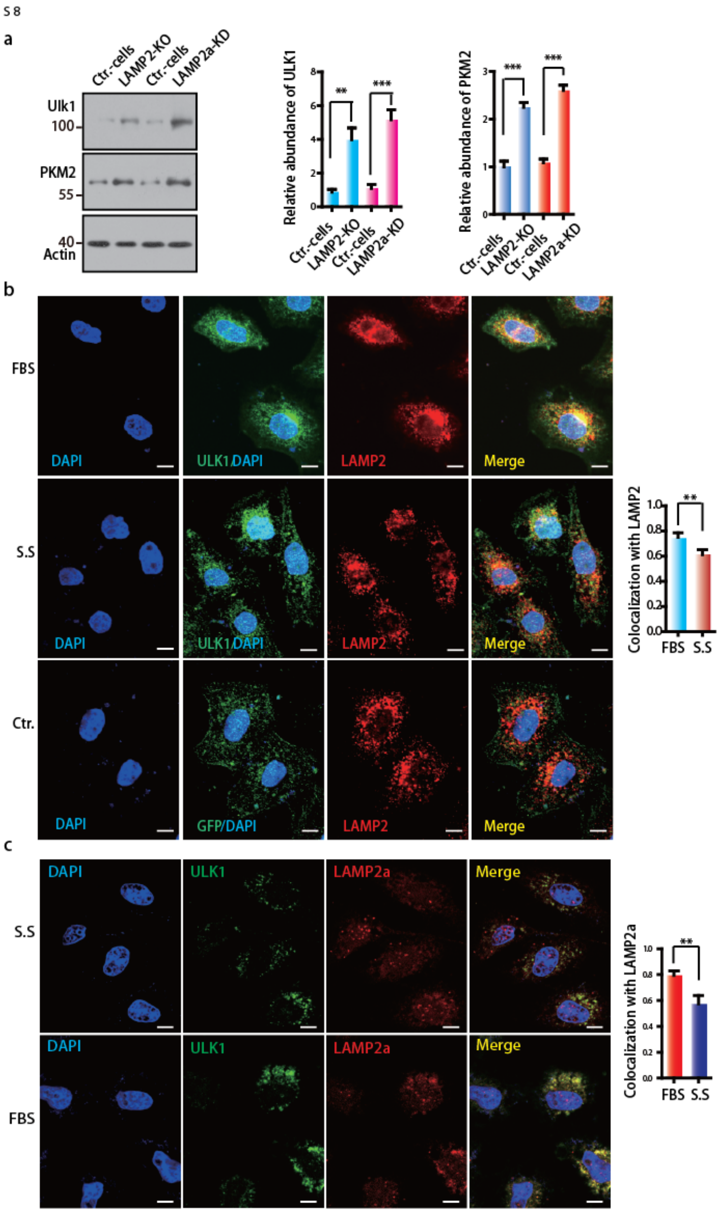

Supplementary Figure 8. ULK1 is linked CMA to macroautophagy. (**a**) CHX(20µg/ml) and MG132(20µM) were used to inhibit the translation process and proteasome function. ULK1s' degradations through lysosome were detected by WB. (**b**) CHX(20µg/ml) and CQ(100nM) were used to inhibit the translation process and lysosome function. ULK1s' degradations through proteasome were detected by WB. (**c**) The Flag-ULK1 was transfected into HEK293 cells and pretreated with CHX(20µg/ml) for 3 hrs,then further incubated with CQ(100nM)+MG132(20µM) or DMSO for indicated time point. The cell lysate was analyzed by WB.(**d**) HOM (Homogenate) and LYS (lysosomes) isolated from Atg5-KD's and ULK1-KO's Hela cells maintained in DMEM media without serum for 16h were subjected to SDS-PAGE and immunoblotted for the indicated proteins. L.E: long exposure; S.E: short exposure (**e**) MTX (Matrices) and MEMB (Membranes) of lysosomes were isolated at the same conditions and analyzed with indicated antibodies.

Supplementary Figure 9

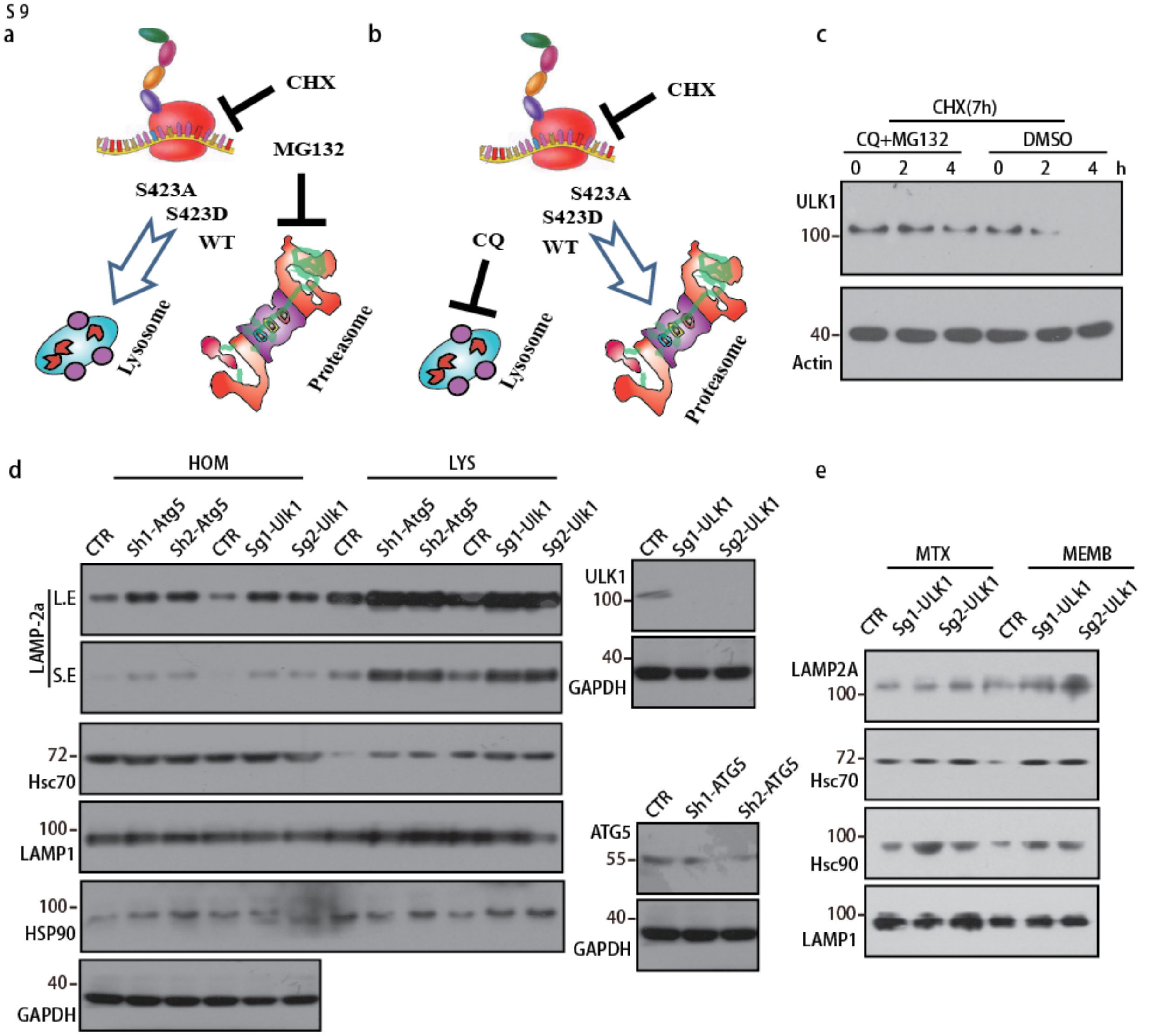

Supplementary Figure 9. ULK1 is degraded through CMA. (**a**) The LAMP2 was knockout through CRISPR/Cas9 system and LAMP2a was knockdown by pGPIZ lentivirus system in HeLa cells. The ULK1 and PKM2 were determined by WB. The quantification was calculated (**b**)Representative images of HeLa cells transfected with GFP-ULK1. The cells were immunostained with anti-LAMP2 antibody (Red) and GFP (Green) and taken the images under confocal microscope. Scale Bar, 5 µM. The represented figure as quantification from a minimum of 20 cells. (**c**) Representative images of HeLa cells transfected with Myc-ULK1. The cells were immunostained with anti-LAMP2a antibody (Red) and anti-Myc antibody (Green) and taken the images under confocal microscope. Scale Bar, 5 µM. The represented figure as quantification from a minimum of 20 cells. All values are means ± SEM of three independent experiments. Student's t test (unpaired); **p<0.01, **p< 0.001. S.S.: serum starvation

Supplementary Figure 10

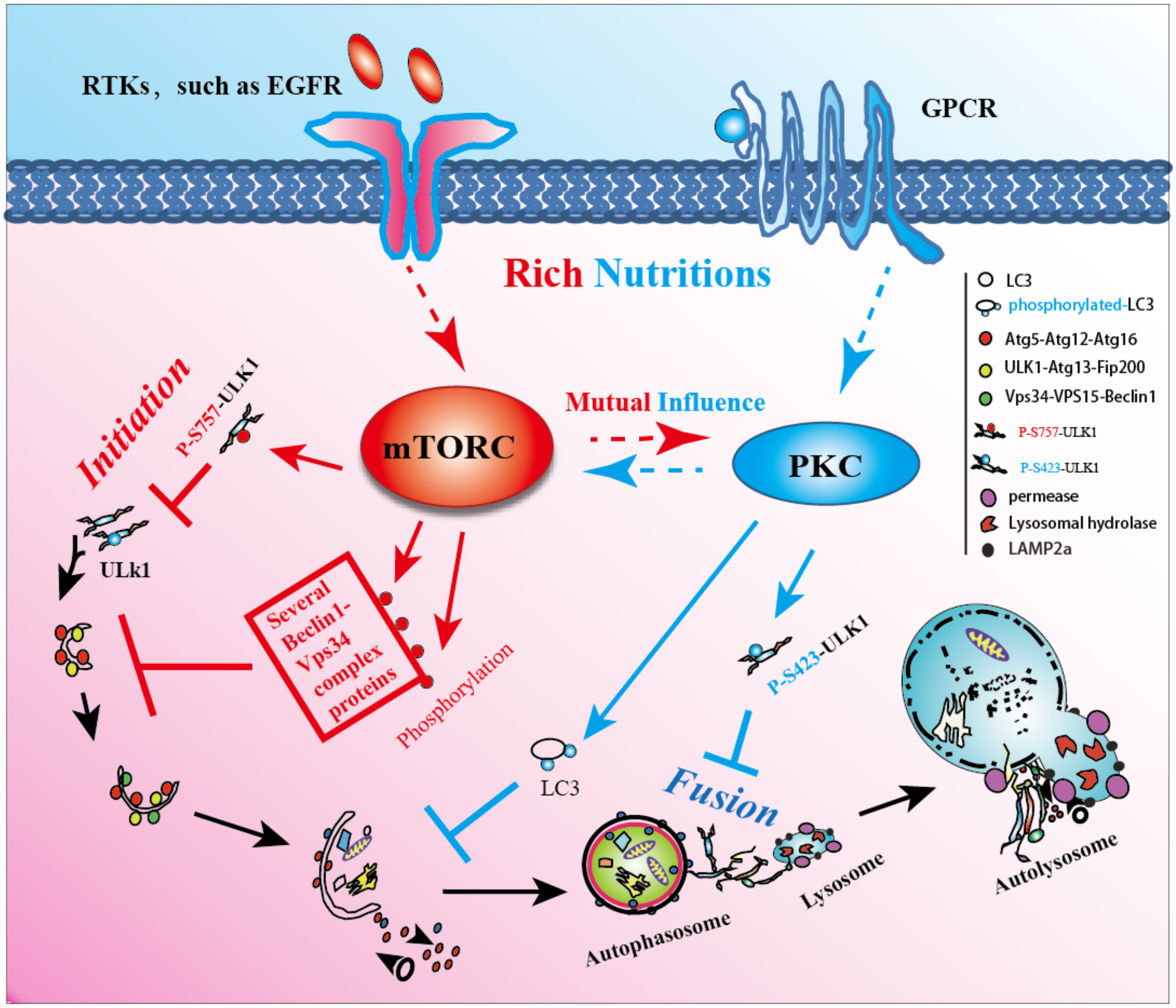

Supplementary Figure 10. Autophagy is regulated by mTOR and PKC. The mechanism of mTORC1 and PKC regulates autophagy at different stages.

Original and Uncropped Blots of Figure 1

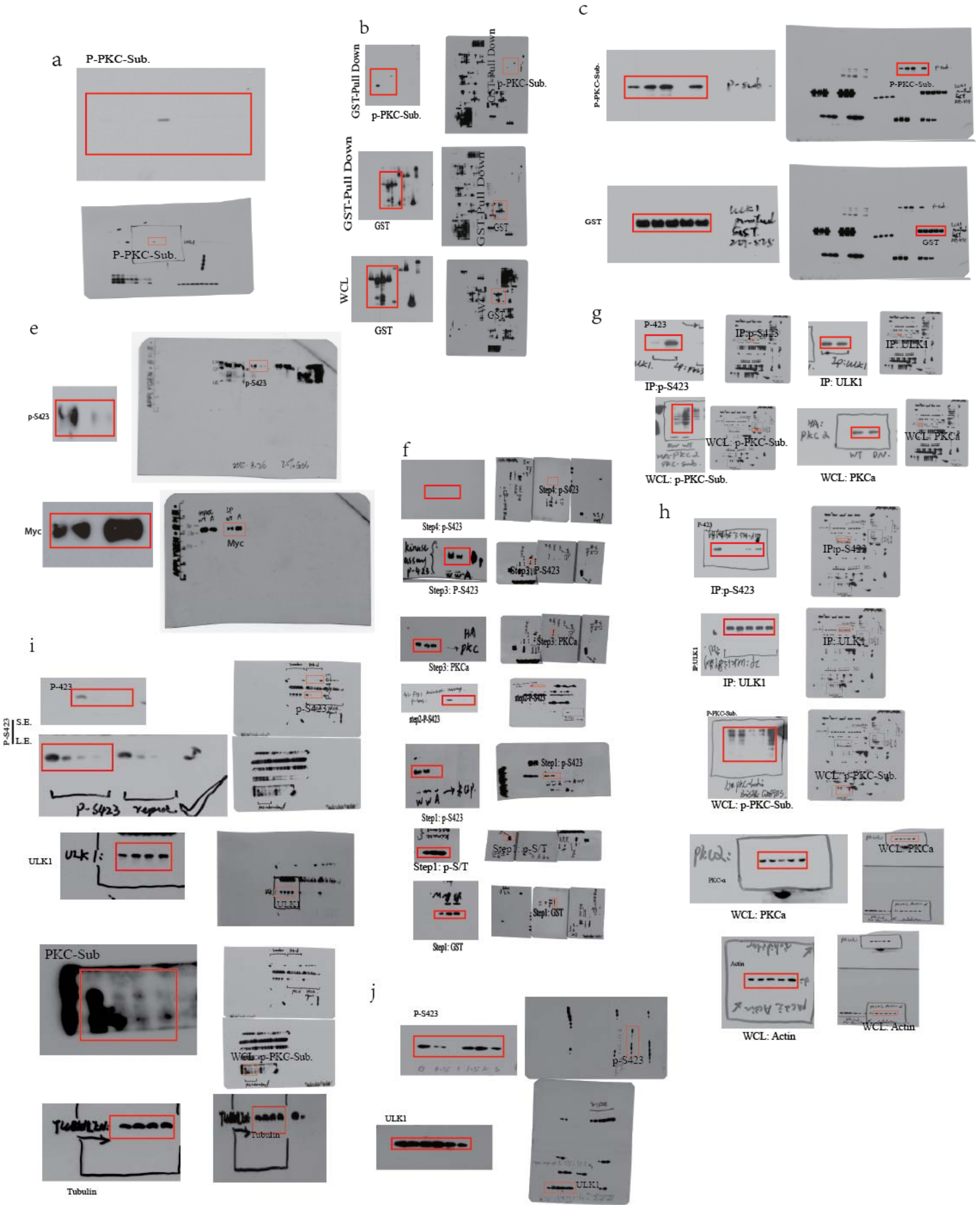

Original and Uncropped Blots of Figure 2

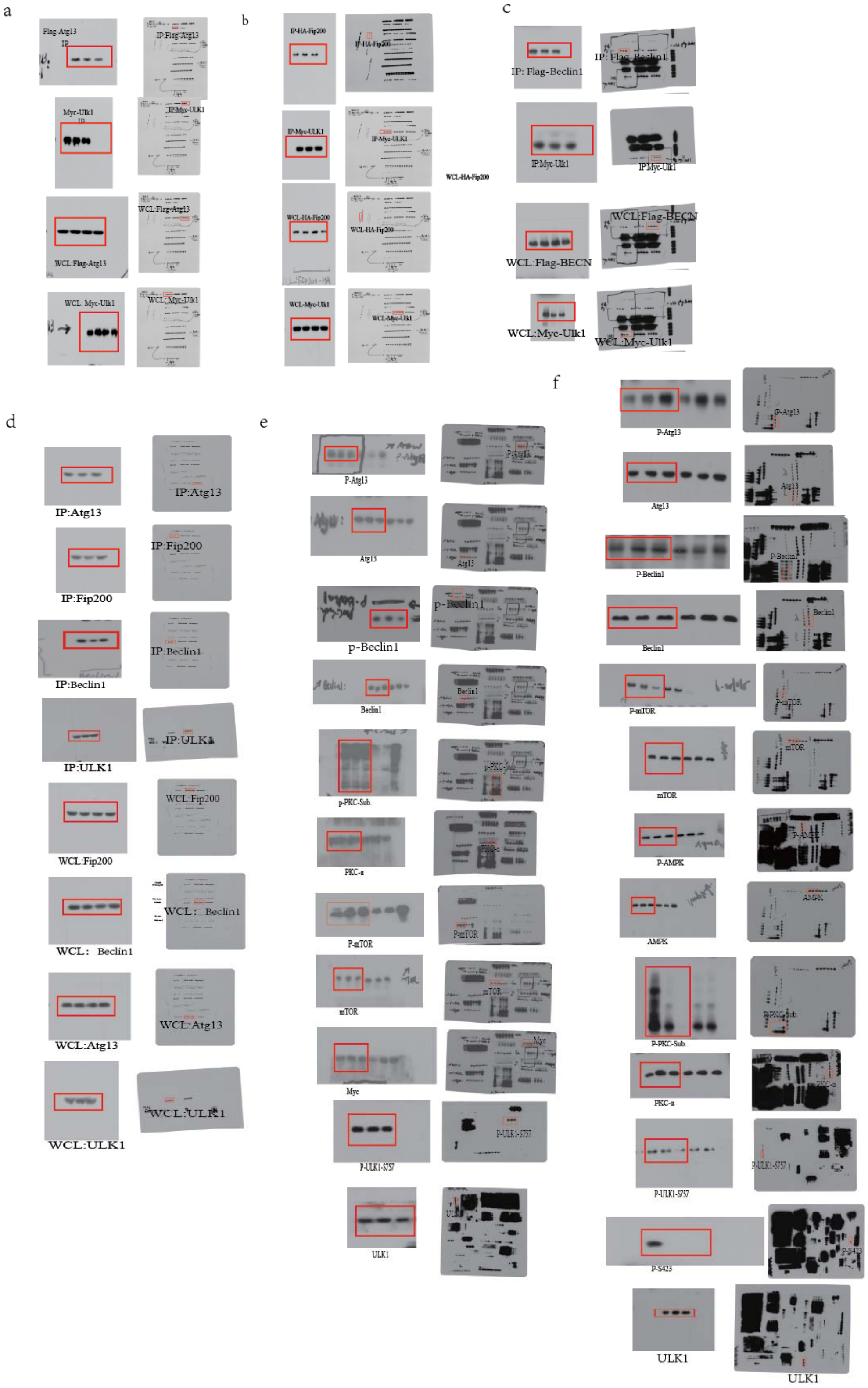

Original and Uncropped Blots of Figure 3

a

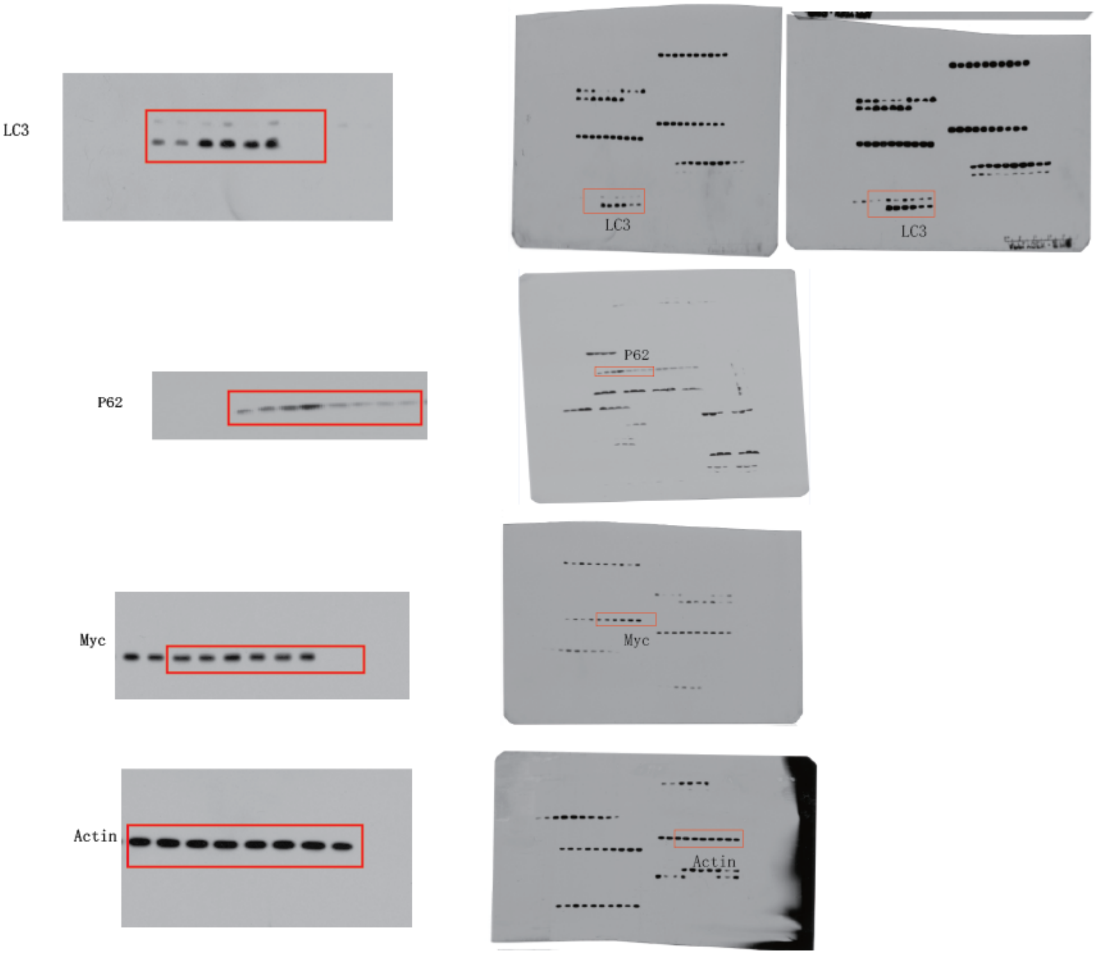

Original and Uncropped Blots of Figure 4

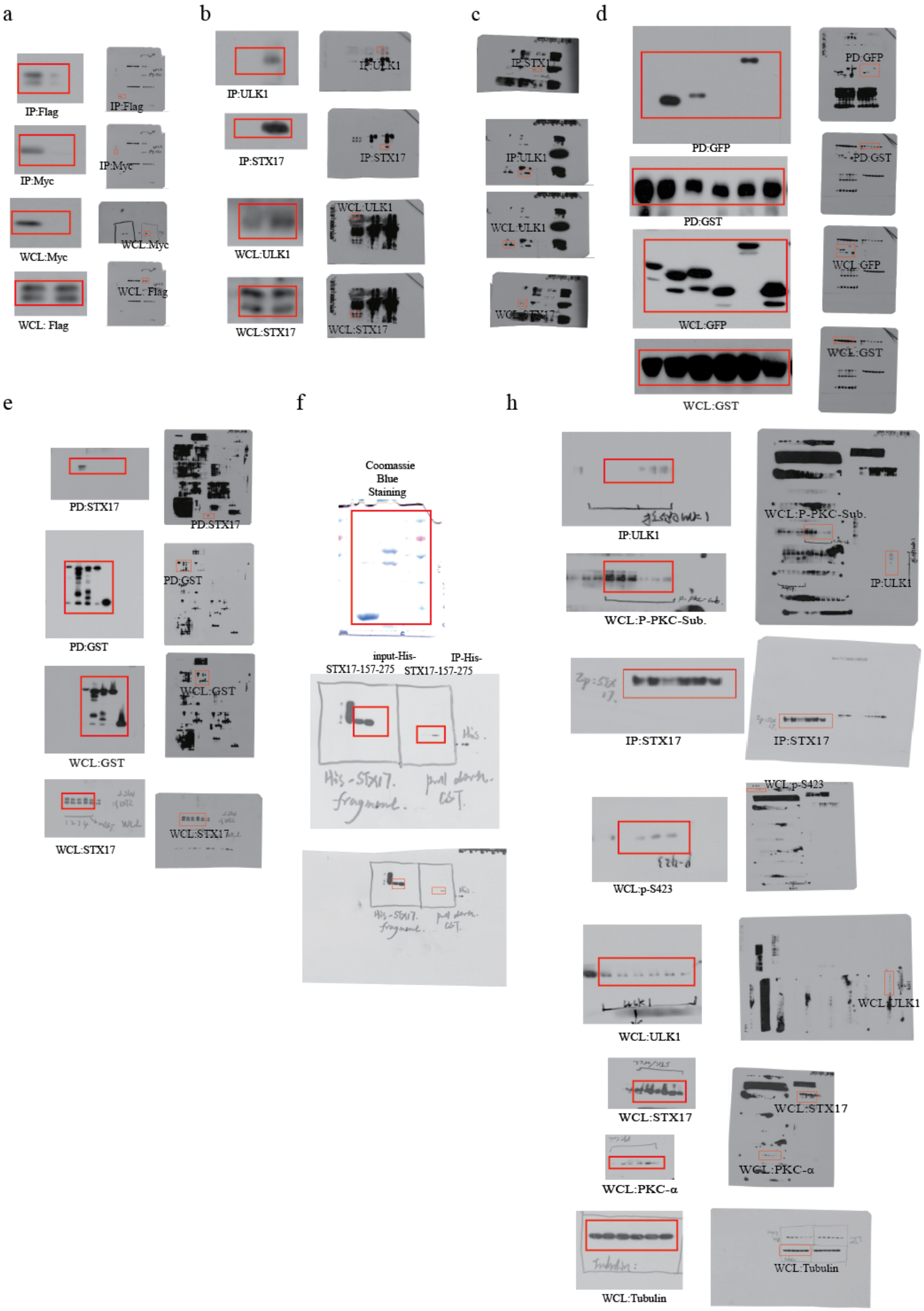

Original and Uncropped Blots of Figure 5

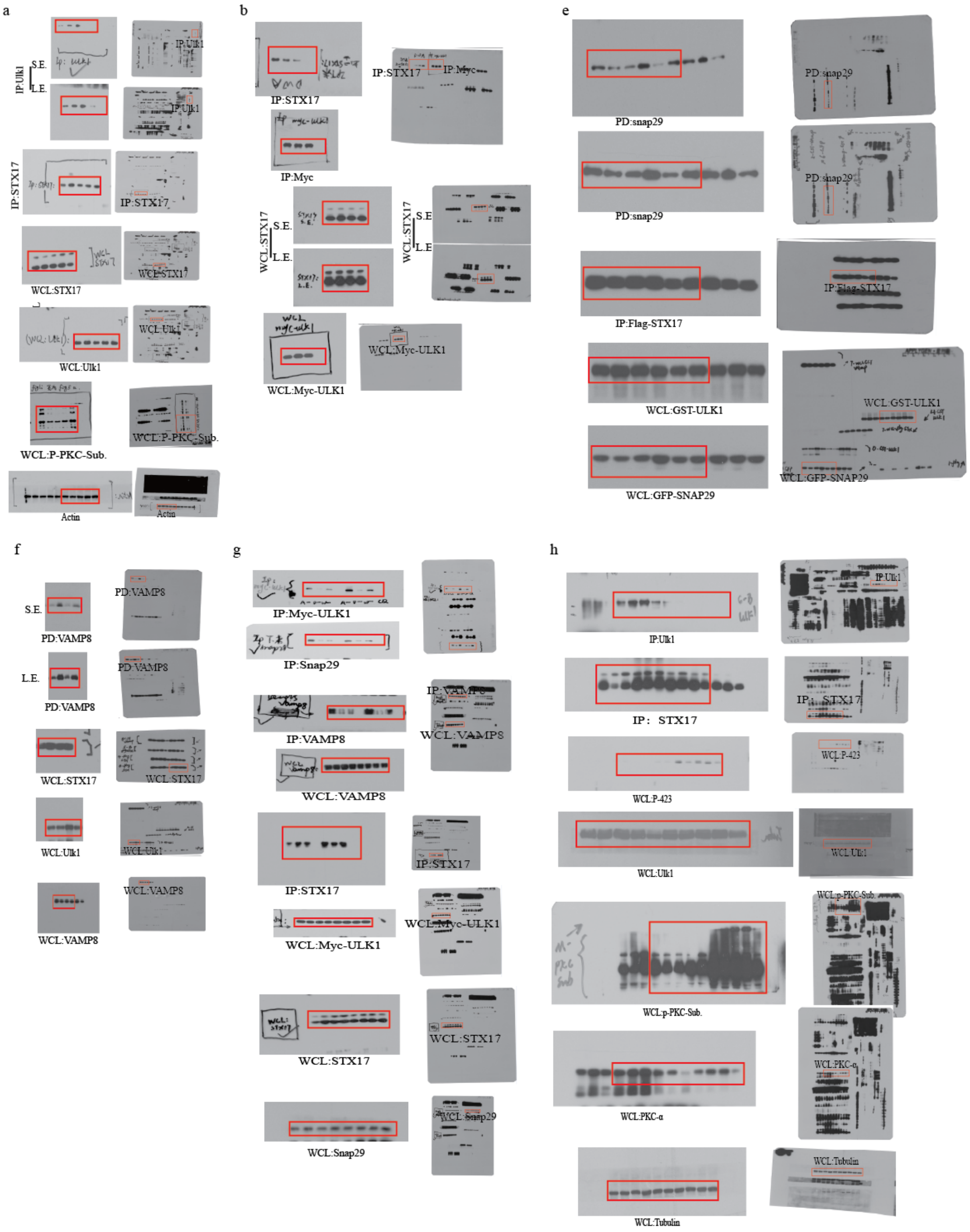

Original and Uncropped Blots of Figure 6

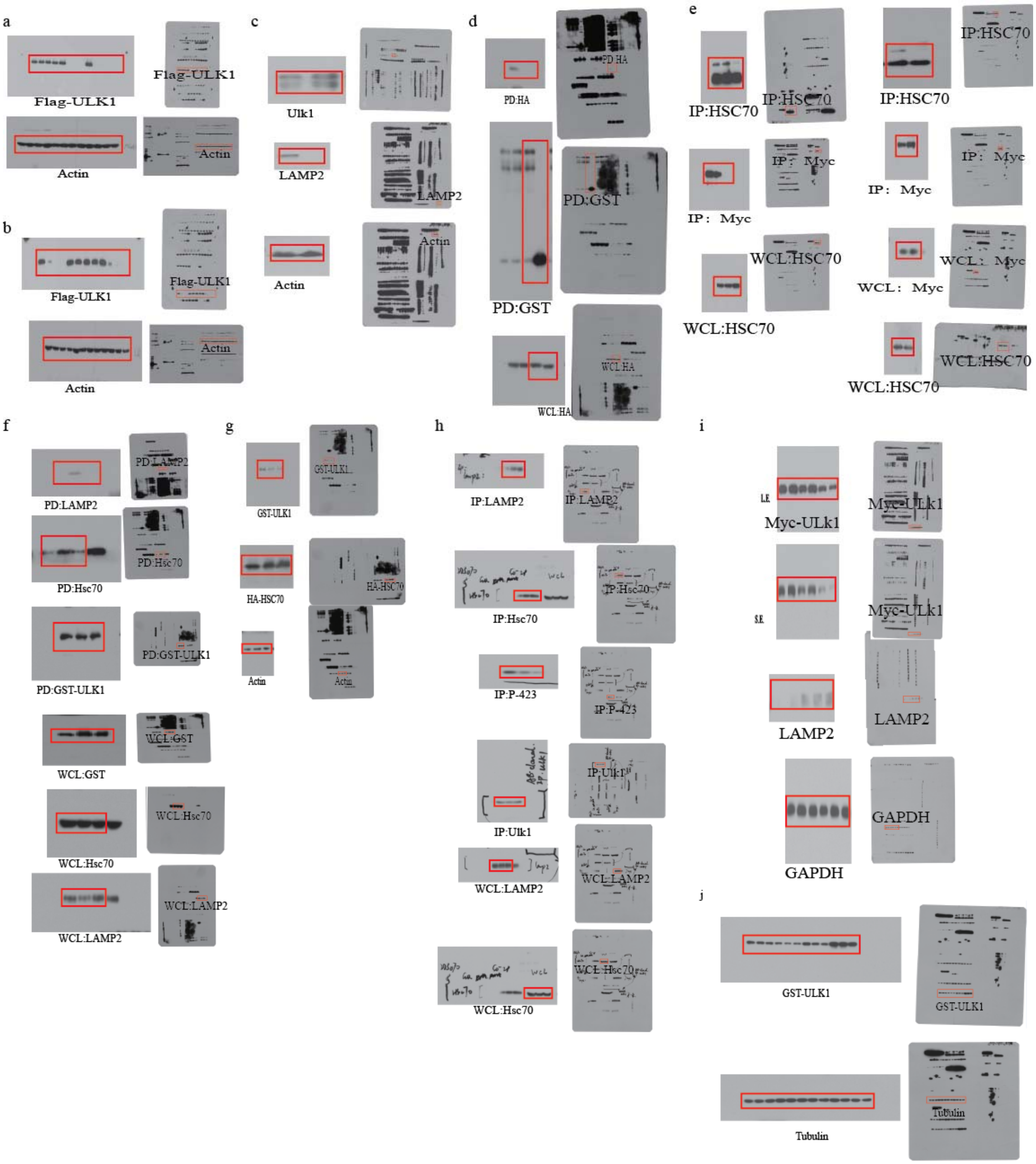

Original and Uncropped Blots of Supplementary Figure 1

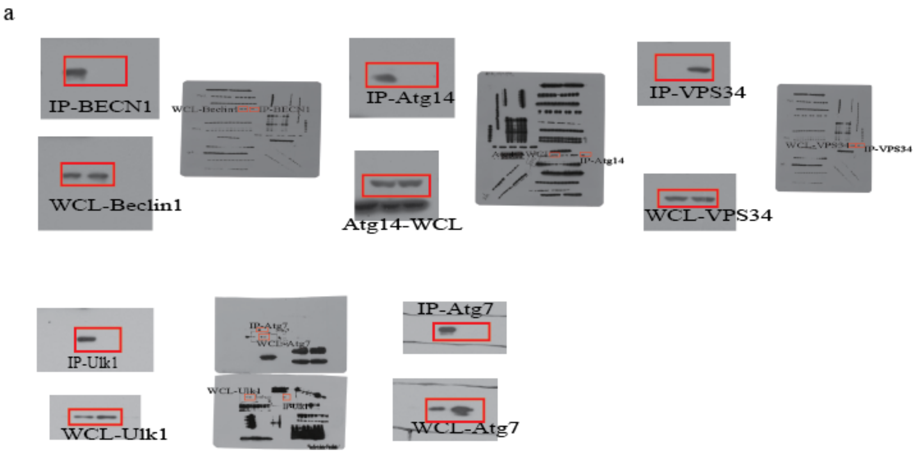

Original and Uncropped Blots of Supplementary Figure 2

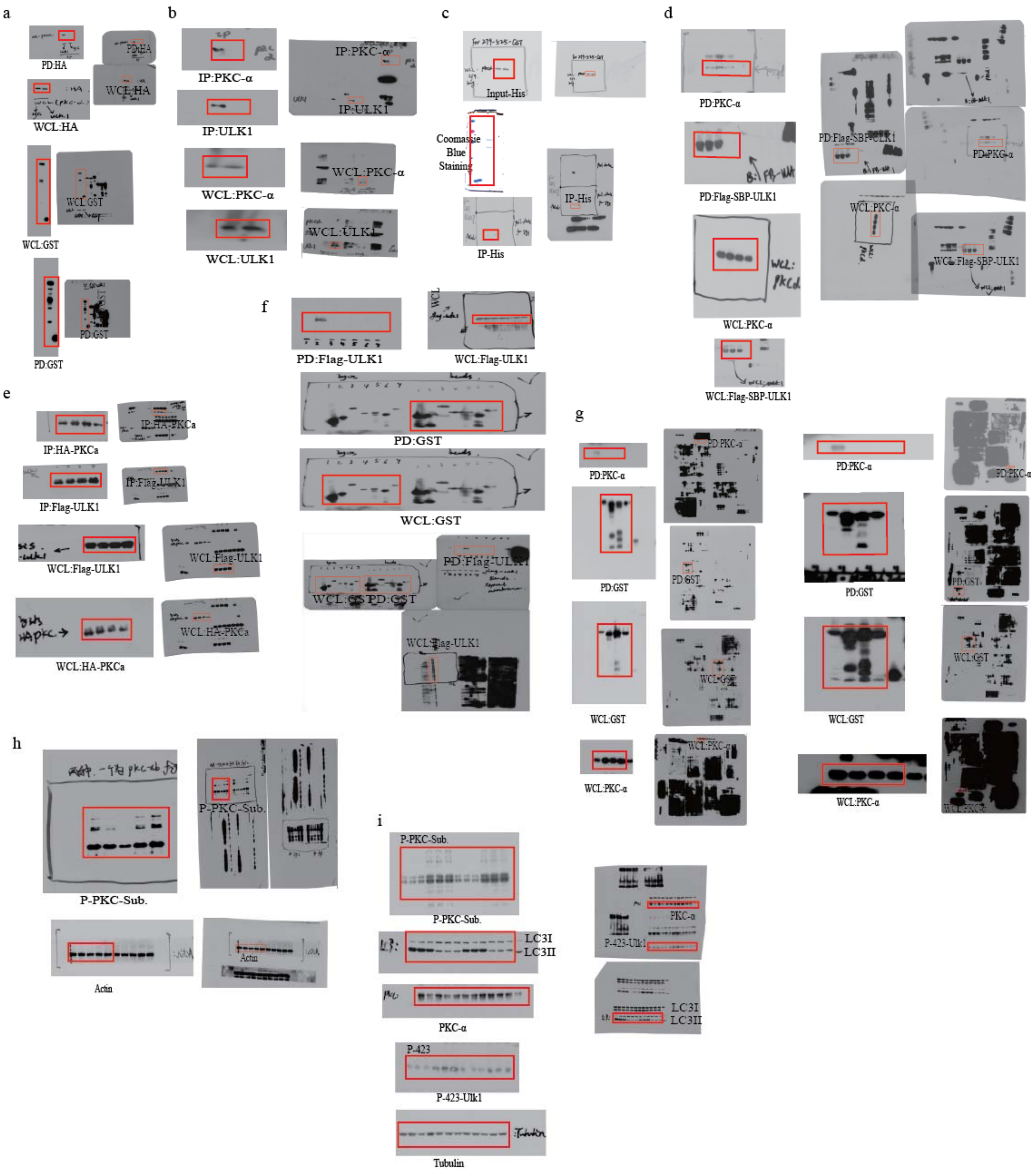

Original and Uncropped Blots of Supplementary Figure 3

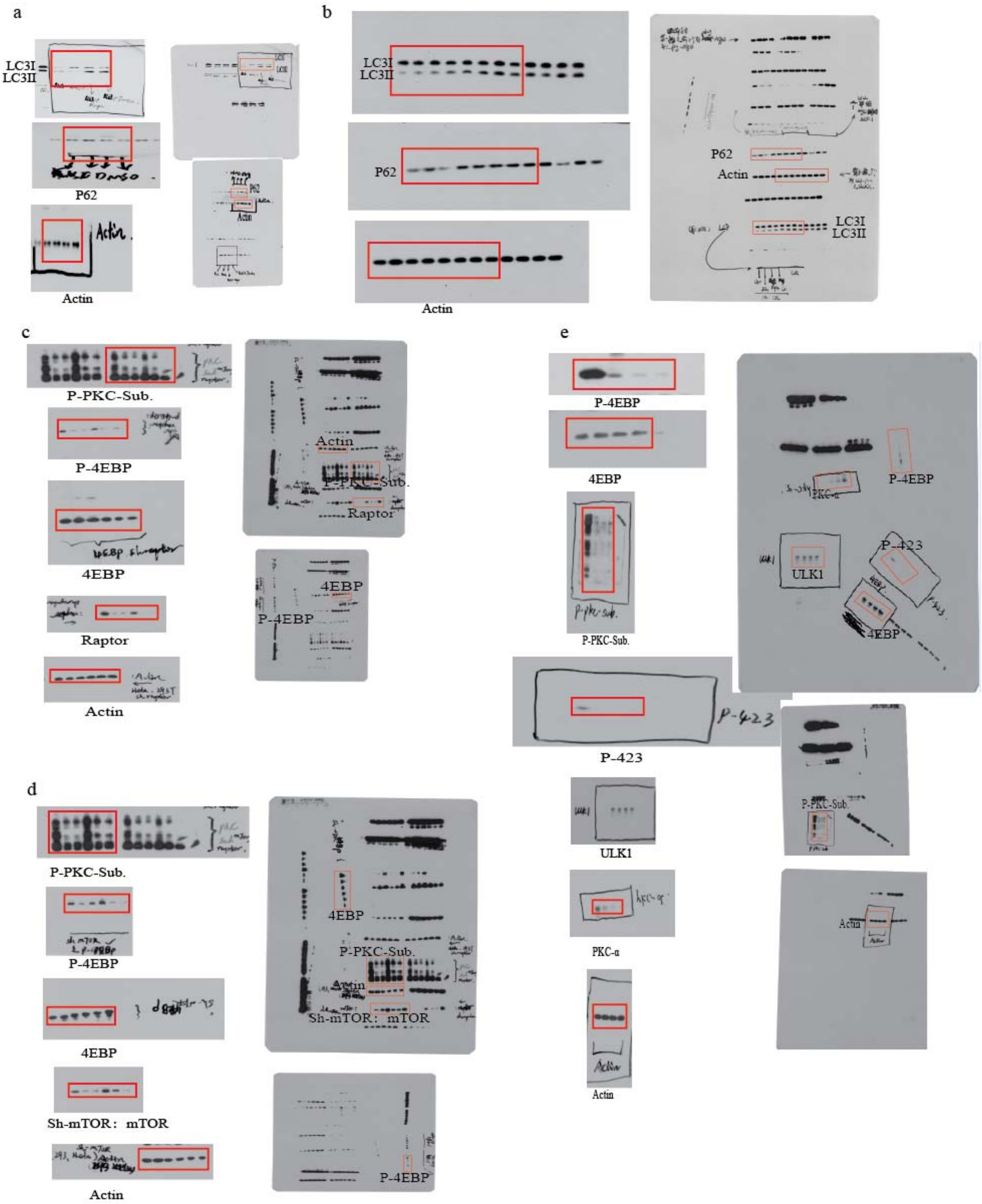

Original and Uncropped Blots of Supplementary Figure 4

a

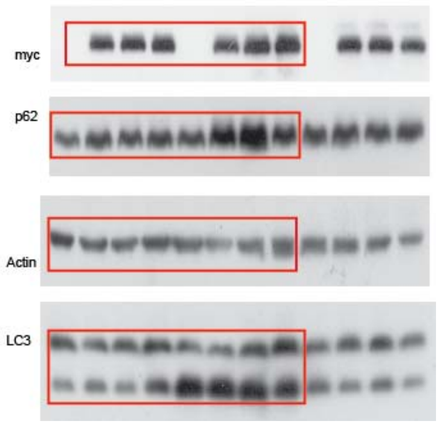

Original and Uncropped Blots of Supplementary Figure 5

C

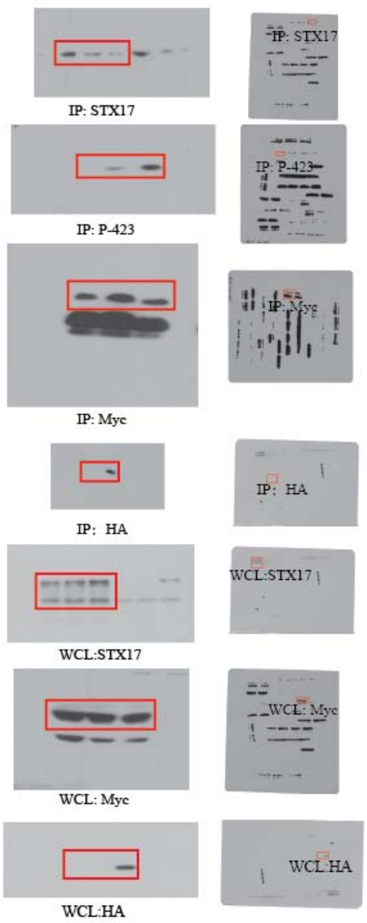

Original and Uncropped Blots of Supplementary Figure 7

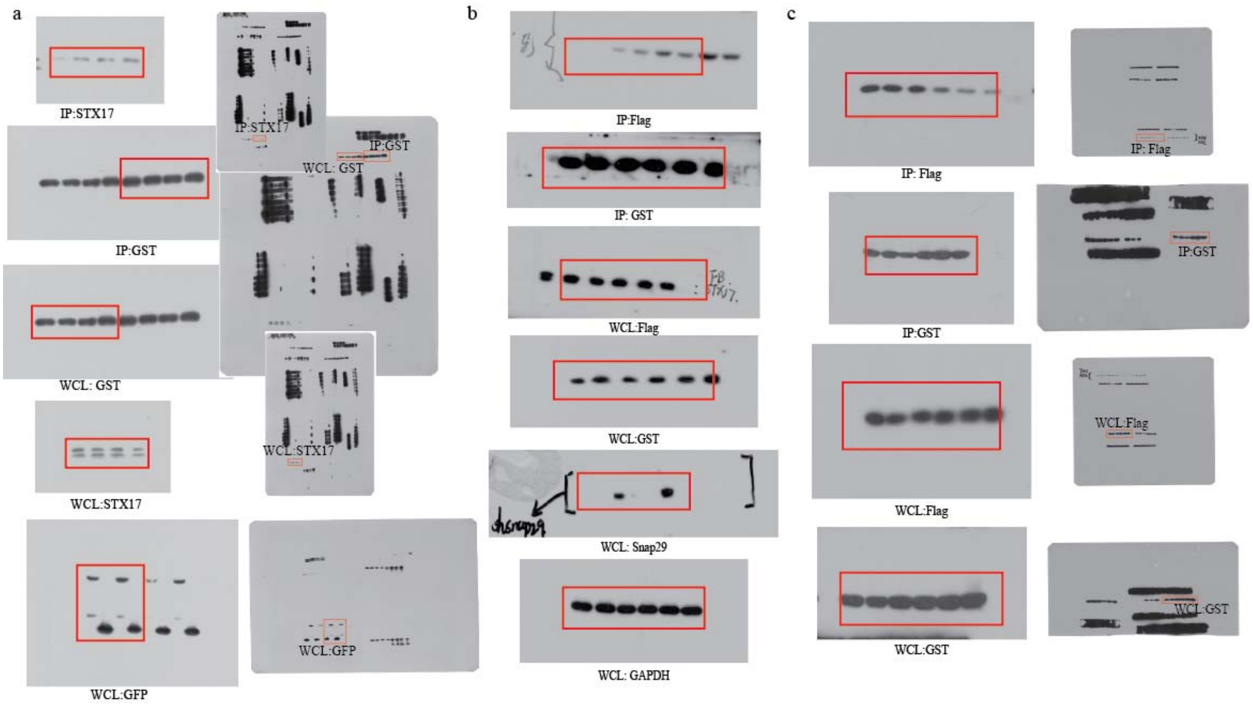

Original and Uncropped Blots of Supplementary Figure 8

a

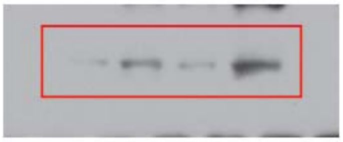

ULK1

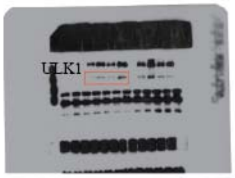

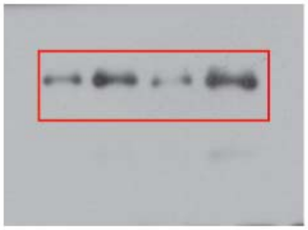

PKM2

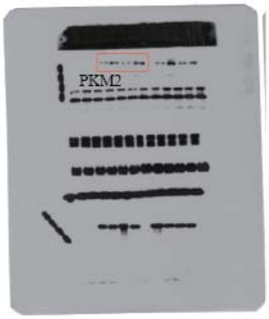

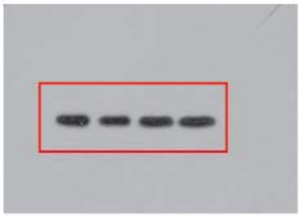

Actin

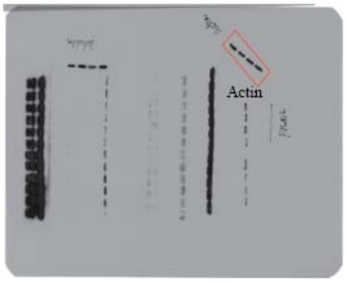

Original and Uncropped Blots of Supplementary Figure 9

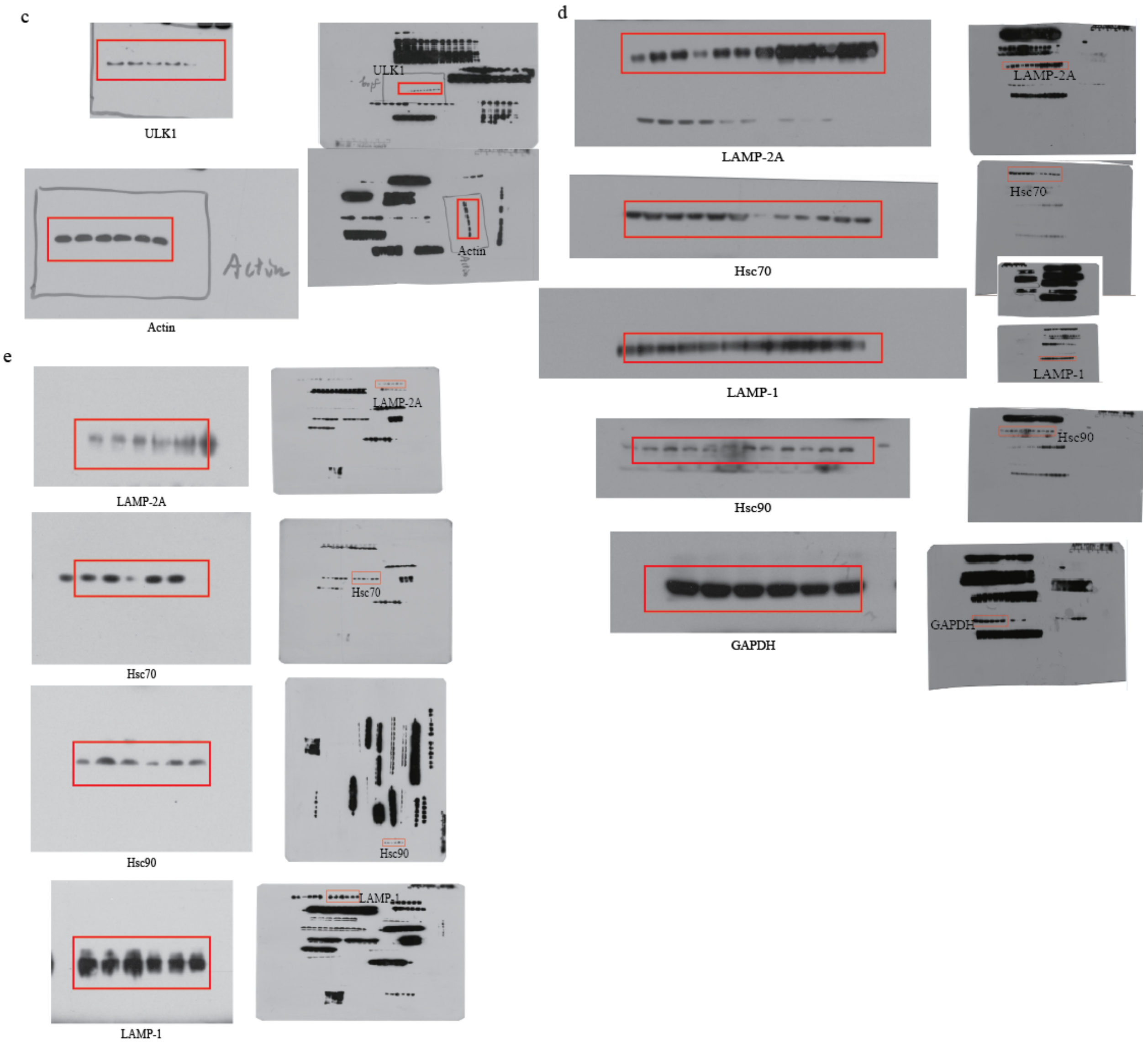

Supplement: Supplementary file 1 — Supplementary Information [file 41467_2018_5449_MOESM1_ESM.pdf]
